# 2-[(3,5-Diphenyl-1*H*-pyrazol-1-yl)meth­yl]pyridine

**DOI:** 10.1107/S1600536812011804

**Published:** 2012-03-24

**Authors:** Ilia A. Guzei, Teddy T. Okemwa, Stephen O. Ojwach

**Affiliations:** a Department of Chemistry, University of Wisconsin-Madison, 1101 University Ave, Madison, WI 53706, USA; b Department of Chemistry, Maseno University, PO Box 333, Maseno 40105, Kenya; c School of Chemistry and Physics, Pietermaritzburg Campus, University of KwaZulu-Natal, Private Bag X01, Scottsville 3209, South Africa

## Abstract

The title compound, C_21_H_17_N_3_, crystallizes with the phenyl ring in the 3-position coplanar with the pyrazole ring within 4.04 (5)°, whereas the phenyl ring in the 5-position forms a dihedral angle of 50.22 (3)° with the pyrazole ring. There is no ambiguity regarding the position of pyridine N atom, which could have exhibited disorder between the *ortho* positions of the ring.

## Related literature

For pyrazole coordination, see: Trofimenko (1993 ▶); Mukherjee (2000 ▶). For our investigation of pyrazolyl-based transition metal complexes as catalysts for olefin transformations, see: Ojwach *et al.* (2009 ▶); Ojwach & Darkwa (2010 ▶). For bond-length data, see: Allen (2002 ▶); Bruno *et al.* (2002 ▶).
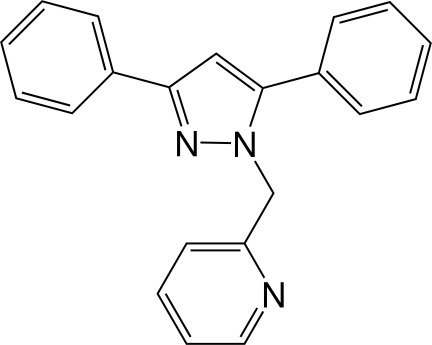


## Experimental

### 

#### Crystal data

C_21_H_17_N_3_
*M* *_r_* = 311.38
Monoclinic, 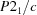

*a* = 12.5776 (8) Å
*b* = 16.531 (1) Å
*c* = 7.9421 (5) Å
β = 97.759 (1)°
*V* = 1636.21 (18) Å^3^
*Z* = 4
Mo *K*α radiation
μ = 0.08 mm^−1^
*T* = 100 K
0.54 × 0.43 × 0.18 mm

#### Data collection

Bruker SMART APEXII area-detector diffractometer
Absorption correction: analytical (*SADABS*; Bruker, 2010 ▶) *T*_min_ = 0.960, *T*_max_ = 0.986
18826 measured reflections
4075 independent reflections
3711 reflections with *I* > 2σ(*I*)
*R*_int_ = 0.024

#### Refinement

*R*[*F* ^2^ > 2σ(*F* ^2^)] = 0.037
*wR*(*F* ^2^) = 0.098
*S* = 1.02
4075 reflections
217 parameters
H-atom parameters constrained
Δρ_max_ = 0.31 e Å^−3^
Δρ_min_ = −0.21 e Å^−3^

### 

Data collection: *APEX2* (Bruker, 2010 ▶); cell refinement: *SAINT-Plus* (Bruker, 2010 ▶); data reduction: *SAINT-Plus*; program(s) used to solve structure: *SHELXTL* (Sheldrick, 2008 ▶); program(s) used to refine structure: *SHELXTL* and *OLEX2* (Dolomanov *et al.*, 2009 ▶); molecular graphics: *SHELXTL*; software used to prepare material for publication: *SHELXTL*, *publCIF* (Westrip, 2010 ▶) and *modiCIFer* (Guzei, 2011 ▶).

## Supplementary Material

Crystal structure: contains datablock(s) global, I. DOI: 10.1107/S1600536812011804/zj2063sup1.cif

Structure factors: contains datablock(s) I. DOI: 10.1107/S1600536812011804/zj2063Isup2.hkl

Supplementary material file. DOI: 10.1107/S1600536812011804/zj2063Isup3.cml

Additional supplementary materials:  crystallographic information; 3D view; checkCIF report

## Figures and Tables

**Fig. 1.**
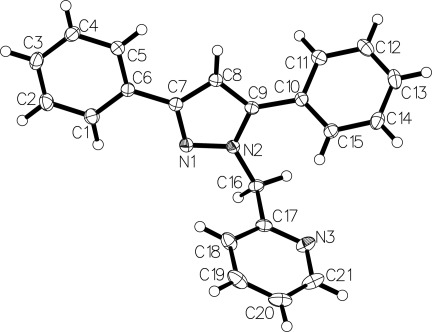
Molecular structure of (I). The thermal ellipsoids are shown at 50% probability level.

## supplementary crystallographic information

### Comment

The coordination chemistry of pyrazolyl ligands with late transition metals has been a subject of numerous invesitigations over the past decades. This is in part due to the ability of the pyrazolyl ligands to display various coordination modes in metal complexes suitable for a wide range of applications (Trofimenko, 1993; Mukherjee, 2000). One such area where pyrazolyl metal complexes have found useful application is in their use as catalysts in various olefin transformations (Ojwach & Darkwa, 2010). As part of our investigation of pyrazolyl-based transition metal complexes as catalysts for olefin transformations (Ojwach *et al.*, 2009 and references therein), we isolated the title compound, (I), Scheme 1, during an attempt to prepare crystals of the zinc complex of 2-(3,5-diphenylpyrazol-1-ylmethyl)pyridine. All bond distances and angles in (I) are within the expected ranges (Bruno *et al.*, 2002). The molecules of (I) pack in columns along the crystallographic *a* axis forming a herring-bone pattern. In (I), the C1 phenyl ring is coplanar with the pyrazole ring within 4.04 (5)°, whereas the C10 phenyl ring forms a 50.22 (3)° dihedral angle with the pyrazole ring. The difference is undoubtedly due to steric conflict between a methylenepyridine at atom N2 and the C10 phenyl ring. A potential problem of this structural investigation was identification of the position of atom N3 which could occupy either of the *ortho* positions in the C17 six-membered ring. In fact when this compound serves as a bidentate ligand the pyridine N atom is on the same side with the pyrazole atom N1. This is not observed in (I). The data quality was sufficiently high to allow unequivocal identification of the position of the N atom in the pyridine ring. An incorrect placement of this atom at the C18 position results in dramatically worse numerical refinement indicators.

### Experimental

To a solution of 2-(3,5-diphenylpyrazol-1-ylmethyl)pyridine (0.10 g, 0.32 mmol) in methanol (10 ml), was added a solution of Zn(Ac)_2_ (0.06 g, 0.32 m mol) in methanol (10 ml) at RT. The clear solution was stirred for 24 h then the solvent was slowly evaporated to afford a white solid. Yield: 0.13 g (81%). Recrystallization of this complex yielde crystals of (I).

### Refinement

All H-atoms were placed in idealized locations and refined as riding with appropriate thermal displacement coefficients *U*_iso_(H) = 1.2 times *U*_eq_(bearing atom).

### Crystal data

**Table:**

C_21_H_17_N_3_	*F*(000) = 656
*M**_r_* = 311.38	*D*_x_ = 1.264 Mg m^−^^3^
Monoclinic, *P*2_1_/*c*	Mo *K*α radiation, λ = 0.71073 Å
Hall symbol: -P 2ybc	Cell parameters from 999 reflections
*a* = 12.5776 (8) Å	θ = 1.6–28.3°
*b* = 16.531 (1) Å	µ = 0.08 mm^−^^1^
*c* = 7.9421 (5) Å	*T* = 100 K
β = 97.759 (1)°	Block, colourless
*V* = 1636.21 (18) Å^3^	0.54 × 0.43 × 0.18 mm
*Z* = 4	

### Data collection

**Table:**

Bruker SMART APEXII area-detector diffractometer	4075 independent reflections
Radiation source: fine-focus sealed tube	3711 reflections with *I* > 2σ(*I*)
Mirror optics monochromator	*R*_int_ = 0.024
0.50° ω and 0.5° φ scans	θ_max_ = 28.3°, θ_min_ = 1.6°
Absorption correction: analytical (*SADABS*; Bruker, 2010)	*h* = −16→16
*T*_min_ = 0.960, *T*_max_ = 0.986	*k* = −22→21
18826 measured reflections	*l* = −10→10

### Refinement

**Table:**

Refinement on *F*^2^	Primary atom site location: structure-invariant direct methods
Least-squares matrix: full	Secondary atom site location: difference Fourier map
*R*[*F*^2^ > 2σ(*F*^2^)] = 0.037	Hydrogen site location: inferred from neighbouring sites
*wR*(*F*^2^) = 0.098	H-atom parameters constrained
*S* = 1.02	*w* = 1/[σ^2^(*F*_o_^2^) + (0.0482*P*)^2^ + 0.5784*P*] where *P* = (*F*_o_^2^ + 2*F*_c_^2^)/3
4075 reflections	(Δ/σ)_max_ < 0.001
217 parameters	Δρ_max_ = 0.31 e Å^−^^3^
0 restraints	Δρ_min_ = −0.21 e Å^−^^3^

### Special details

**Table:**

Geometry. All e.s.d.'s (except the e.s.d. in the dihedral angle between two l.s. planes) are estimated using the full covariance matrix. The cell e.s.d.'s are taken into account individually in the estimation of e.s.d.'s in distances, angles and torsion angles; correlations between e.s.d.'s in cell parameters are only used when they are defined by crystal symmetry. An approximate (isotropic) treatment of cell e.s.d.'s is used for estimating e.s.d.'s involving l.s. planes.
Refinement. Refinement of *F*^2^ against ALL reflections. The weighted *R*-factor *wR* and goodness of fit *S* are based on *F*^2^, conventional *R*-factors *R* are based on *F*, with *F* set to zero for negative *F*^2^. The threshold expression of *F*^2^ > σ(*F*^2^) is used only for calculating *R*-factors(gt) *etc*. and is not relevant to the choice of reflections for refinement. *R*-factors based on *F*^2^ are statistically about twice as large as those based on *F*, and *R*- factors based on ALL data will be even larger.

### Fractional atomic coordinates and isotropic or equivalent isotropic displacement parameters (Å^2^)

**Table:**

	*x*	*y*	*z*	*U*_iso_*/*U*_eq_	
N1	0.56283 (6)	0.11731 (5)	0.87783 (10)	0.01783 (16)	
N2	0.46026 (6)	0.14515 (5)	0.86210 (10)	0.01640 (16)	
N3	0.21604 (7)	0.03283 (6)	0.77928 (11)	0.02588 (19)	
C1	0.79245 (8)	0.10240 (6)	0.88480 (12)	0.02147 (19)	
H1	0.7557	0.0600	0.9334	0.026*	
C2	0.90248 (8)	0.09663 (6)	0.88094 (13)	0.0253 (2)	
H2	0.9402	0.0501	0.9269	0.030*	
C3	0.95788 (8)	0.15812 (6)	0.81065 (13)	0.0247 (2)	
H3	1.0329	0.1537	0.8078	0.030*	
C4	0.90209 (8)	0.22622 (6)	0.74455 (13)	0.0231 (2)	
H4	0.9393	0.2688	0.6973	0.028*	
C5	0.79200 (7)	0.23231 (6)	0.74737 (12)	0.01950 (18)	
H5	0.7546	0.2789	0.7013	0.023*	
C6	0.73581 (7)	0.17054 (5)	0.81721 (11)	0.01686 (18)	
C7	0.61924 (7)	0.17777 (5)	0.81945 (11)	0.01638 (18)	
C8	0.55255 (7)	0.24429 (5)	0.76702 (11)	0.01741 (18)	
H8	0.5731	0.2940	0.7209	0.021*	
C9	0.45102 (7)	0.22172 (5)	0.79693 (11)	0.01610 (17)	
C10	0.35049 (7)	0.26845 (5)	0.77034 (11)	0.01638 (18)	
C11	0.34982 (8)	0.34795 (6)	0.83071 (12)	0.02013 (19)	
H11	0.4130	0.3702	0.8926	0.024*	
C12	0.25718 (9)	0.39466 (6)	0.80070 (13)	0.0247 (2)	
H12	0.2572	0.4485	0.8425	0.030*	
C13	0.16471 (8)	0.36260 (7)	0.70964 (14)	0.0266 (2)	
H13	0.1016	0.3946	0.6886	0.032*	
C14	0.16455 (8)	0.28366 (6)	0.64930 (13)	0.0236 (2)	
H14	0.1013	0.2618	0.5869	0.028*	
C15	0.25656 (7)	0.23660 (6)	0.67999 (11)	0.01905 (18)	
H15	0.2558	0.1825	0.6395	0.023*	
C16	0.37702 (7)	0.09527 (5)	0.92095 (11)	0.01754 (18)	
H16A	0.3216	0.1312	0.9579	0.021*	
H16B	0.4092	0.0640	1.0214	0.021*	
C17	0.32303 (8)	0.03679 (5)	0.78899 (11)	0.01818 (18)	
C18	0.38216 (9)	−0.01146 (6)	0.69164 (12)	0.0251 (2)	
H18	0.4579	−0.0062	0.7007	0.030*	
C19	0.32785 (10)	−0.06739 (6)	0.58106 (14)	0.0309 (2)	
H19	0.3660	−0.1012	0.5131	0.037*	
C20	0.21751 (10)	−0.07312 (6)	0.57135 (13)	0.0304 (2)	
H20	0.1784	−0.1113	0.4979	0.036*	
C21	0.16543 (9)	−0.02194 (7)	0.67126 (14)	0.0309 (2)	
H21	0.0895	−0.0257	0.6632	0.037*	

### Atomic displacement parameters (Å^2^)

**Table:**

	*U*^11^	*U*^22^	*U*^33^	*U*^12^	*U*^13^	*U*^23^
N1	0.0172 (4)	0.0175 (4)	0.0190 (4)	0.0006 (3)	0.0033 (3)	0.0000 (3)
N2	0.0163 (4)	0.0153 (4)	0.0181 (3)	−0.0003 (3)	0.0038 (3)	0.0003 (3)
N3	0.0255 (4)	0.0282 (4)	0.0249 (4)	−0.0098 (3)	0.0068 (3)	−0.0030 (3)
C1	0.0212 (4)	0.0211 (4)	0.0213 (4)	0.0011 (3)	−0.0001 (3)	0.0026 (3)
C2	0.0212 (5)	0.0253 (5)	0.0276 (5)	0.0053 (4)	−0.0037 (4)	0.0003 (4)
C3	0.0156 (4)	0.0300 (5)	0.0275 (5)	0.0009 (4)	−0.0006 (3)	−0.0070 (4)
C4	0.0191 (4)	0.0230 (5)	0.0276 (5)	−0.0031 (4)	0.0052 (4)	−0.0041 (4)
C5	0.0183 (4)	0.0176 (4)	0.0228 (4)	0.0006 (3)	0.0034 (3)	−0.0015 (3)
C6	0.0167 (4)	0.0178 (4)	0.0159 (4)	0.0002 (3)	0.0013 (3)	−0.0023 (3)
C7	0.0178 (4)	0.0166 (4)	0.0146 (4)	−0.0003 (3)	0.0019 (3)	−0.0009 (3)
C8	0.0179 (4)	0.0159 (4)	0.0186 (4)	−0.0003 (3)	0.0030 (3)	0.0008 (3)
C9	0.0183 (4)	0.0148 (4)	0.0153 (4)	−0.0004 (3)	0.0026 (3)	−0.0006 (3)
C10	0.0170 (4)	0.0175 (4)	0.0155 (4)	0.0009 (3)	0.0052 (3)	0.0019 (3)
C11	0.0237 (4)	0.0190 (4)	0.0184 (4)	0.0006 (3)	0.0050 (3)	0.0000 (3)
C12	0.0317 (5)	0.0200 (4)	0.0241 (5)	0.0068 (4)	0.0096 (4)	0.0006 (4)
C13	0.0225 (5)	0.0297 (5)	0.0290 (5)	0.0099 (4)	0.0089 (4)	0.0065 (4)
C14	0.0168 (4)	0.0293 (5)	0.0250 (5)	0.0002 (4)	0.0044 (3)	0.0055 (4)
C15	0.0184 (4)	0.0198 (4)	0.0196 (4)	−0.0009 (3)	0.0051 (3)	0.0017 (3)
C16	0.0197 (4)	0.0169 (4)	0.0167 (4)	−0.0031 (3)	0.0049 (3)	0.0000 (3)
C17	0.0240 (4)	0.0144 (4)	0.0161 (4)	−0.0025 (3)	0.0023 (3)	0.0029 (3)
C18	0.0287 (5)	0.0231 (5)	0.0216 (4)	0.0072 (4)	−0.0032 (4)	−0.0028 (4)
C19	0.0445 (6)	0.0221 (5)	0.0232 (5)	0.0092 (4)	−0.0058 (4)	−0.0044 (4)
C20	0.0474 (6)	0.0198 (5)	0.0212 (5)	−0.0094 (4)	−0.0054 (4)	0.0010 (4)
C21	0.0318 (5)	0.0339 (6)	0.0267 (5)	−0.0165 (4)	0.0031 (4)	−0.0005 (4)

### Geometric parameters (Å, º)

**Table:**

N1—C7	1.3440 (12)	C10—C11	1.3994 (13)
N1—N2	1.3595 (10)	C10—C15	1.3997 (12)
N2—C9	1.3664 (11)	C11—C12	1.3913 (13)
N2—C16	1.4584 (11)	C11—H11	0.9500
N3—C17	1.3390 (13)	C12—C13	1.3891 (16)
N3—C21	1.3468 (13)	C12—H12	0.9500
C1—C2	1.3916 (14)	C13—C14	1.3901 (15)
C1—C6	1.4005 (13)	C13—H13	0.9500
C1—H1	0.9500	C14—C15	1.3884 (13)
C2—C3	1.3912 (15)	C14—H14	0.9500
C2—H2	0.9500	C15—H15	0.9500
C3—C4	1.3914 (14)	C16—C17	1.5172 (12)
C3—H3	0.9500	C16—H16A	0.9900
C4—C5	1.3916 (13)	C16—H16B	0.9900
C4—H4	0.9500	C17—C18	1.3936 (14)
C5—C6	1.3985 (13)	C18—C19	1.3895 (14)
C5—H5	0.9500	C18—H18	0.9500
C6—C7	1.4736 (12)	C19—C20	1.3829 (17)
C7—C8	1.4112 (12)	C19—H19	0.9500
C8—C9	1.3814 (12)	C20—C21	1.3838 (17)
C8—H8	0.9500	C20—H20	0.9500
C9—C10	1.4725 (12)	C21—H21	0.9500
			
C7—N1—N2	104.78 (7)	C12—C11—H11	119.8
N1—N2—C9	112.31 (7)	C10—C11—H11	119.8
N1—N2—C16	119.51 (7)	C13—C12—C11	120.03 (9)
C9—N2—C16	128.11 (8)	C13—C12—H12	120.0
C17—N3—C21	117.03 (9)	C11—C12—H12	120.0
C2—C1—C6	120.17 (9)	C12—C13—C14	119.99 (9)
C2—C1—H1	119.9	C12—C13—H13	120.0
C6—C1—H1	119.9	C14—C13—H13	120.0
C3—C2—C1	120.81 (9)	C15—C14—C13	120.20 (9)
C3—C2—H2	119.6	C15—C14—H14	119.9
C1—C2—H2	119.6	C13—C14—H14	119.9
C2—C3—C4	119.22 (9)	C14—C15—C10	120.34 (9)
C2—C3—H3	120.4	C14—C15—H15	119.8
C4—C3—H3	120.4	C10—C15—H15	119.8
C3—C4—C5	120.31 (9)	N2—C16—C17	114.37 (7)
C3—C4—H4	119.8	N2—C16—H16A	108.7
C5—C4—H4	119.8	C17—C16—H16A	108.7
C4—C5—C6	120.72 (9)	N2—C16—H16B	108.7
C4—C5—H5	119.6	C17—C16—H16B	108.7
C6—C5—H5	119.6	H16A—C16—H16B	107.6
C5—C6—C1	118.76 (8)	N3—C17—C18	123.20 (9)
C5—C6—C7	120.16 (8)	N3—C17—C16	115.06 (8)
C1—C6—C7	121.08 (8)	C18—C17—C16	121.68 (9)
N1—C7—C8	111.15 (8)	C19—C18—C17	118.54 (10)
N1—C7—C6	121.08 (8)	C19—C18—H18	120.7
C8—C7—C6	127.76 (8)	C17—C18—H18	120.7
C9—C8—C7	105.38 (8)	C20—C19—C18	119.00 (10)
C9—C8—H8	127.3	C20—C19—H19	120.5
C7—C8—H8	127.3	C18—C19—H19	120.5
N2—C9—C8	106.38 (8)	C19—C20—C21	118.38 (10)
N2—C9—C10	124.60 (8)	C19—C20—H20	120.8
C8—C9—C10	129.01 (8)	C21—C20—H20	120.8
C11—C10—C15	119.03 (8)	N3—C21—C20	123.83 (10)
C11—C10—C9	119.24 (8)	N3—C21—H21	118.1
C15—C10—C9	121.68 (8)	C20—C21—H21	118.1
C12—C11—C10	120.41 (9)		
			
C7—N1—N2—C9	−0.57 (10)	N2—C9—C10—C11	130.54 (9)
C7—N1—N2—C16	−177.56 (7)	C8—C9—C10—C11	−48.00 (13)
C6—C1—C2—C3	0.13 (15)	N2—C9—C10—C15	−52.16 (13)
C1—C2—C3—C4	0.37 (15)	C8—C9—C10—C15	129.30 (10)
C2—C3—C4—C5	−0.61 (15)	C15—C10—C11—C12	−0.24 (13)
C3—C4—C5—C6	0.34 (14)	C9—C10—C11—C12	177.13 (8)
C4—C5—C6—C1	0.16 (14)	C10—C11—C12—C13	−0.30 (14)
C4—C5—C6—C7	179.97 (8)	C11—C12—C13—C14	0.37 (15)
C2—C1—C6—C5	−0.40 (14)	C12—C13—C14—C15	0.11 (15)
C2—C1—C6—C7	179.80 (8)	C13—C14—C15—C10	−0.65 (14)
N2—N1—C7—C8	0.19 (10)	C11—C10—C15—C14	0.71 (13)
N2—N1—C7—C6	179.52 (7)	C9—C10—C15—C14	−176.59 (8)
C5—C6—C7—N1	176.49 (8)	N1—N2—C16—C17	−88.21 (10)
C1—C6—C7—N1	−3.71 (13)	C9—N2—C16—C17	95.33 (11)
C5—C6—C7—C8	−4.31 (14)	C21—N3—C17—C18	1.16 (14)
C1—C6—C7—C8	175.49 (9)	C21—N3—C17—C16	−176.00 (9)
N1—C7—C8—C9	0.23 (10)	N2—C16—C17—N3	−136.02 (8)
C6—C7—C8—C9	−179.04 (8)	N2—C16—C17—C18	46.77 (12)
N1—N2—C9—C8	0.72 (10)	N3—C17—C18—C19	−1.10 (15)
C16—N2—C9—C8	177.40 (8)	C16—C17—C18—C19	175.88 (9)
N1—N2—C9—C10	−178.10 (8)	C17—C18—C19—C20	0.08 (15)
C16—N2—C9—C10	−1.42 (14)	C18—C19—C20—C21	0.77 (16)
C7—C8—C9—N2	−0.55 (9)	C17—N3—C21—C20	−0.23 (16)
C7—C8—C9—C10	178.20 (8)	C19—C20—C21—N3	−0.73 (17)
